# High Methoxyl Pectin Consistently Reduces β‐Carotene Bioaccessibility Across Various Gastrointestinal Digestion Conditions

**DOI:** 10.1002/mnfr.70562

**Published:** 2026-08-05

**Authors:** Anushka Shukla, Thomas Schleeh, Patrick Borel, Charles Desmarchelier, Torsten Bohn

**Affiliations:** ^1^ Nutrition and Health Research Group, Department of Precision Health Luxembourg Institute of Health Strassen Luxembourg; ^2^ Luxembourg Institute of Science and Technology Belval Luxembourg; ^3^ C2VN Aix‐Marseille Univ, INRAE, INSERM Marseille France; ^4^ Institut Universitaire de France (IUF) Paris France

**Keywords:** bioavailability, carotenoids, dietary fibers, high methoxyl pectin, in vitro digestion, static digestion

## Abstract

Dietary fiber could inhibit β‐carotene bioaccessibility by restricting its release from the food matrix, interfering with digestive enzyme activities, binding bile salts, or modifying viscosity and other physicochemical properties of the digesta. In this study, we investigated whether high methoxyl pectin (HMP), a soluble dietary fiber found in fruits/vegetables and an additive for the food industry would impact β‐carotene bioaccessibility under various physiological digestive conditions, following the INFOGEST gastrointestinal model. Concentrations of pancreatin plus bile salts and shear forces (simulated by varying water bath rounds/min. and glass bead addition) were modified in the presence (1.15 mg/mL digesta) and absence of HMP. Endpoints measured in the digesta included β‐carotene bioaccessibility, surface tension, viscosity, micelle size, zeta potential, and triglyceride lipolysis. Adding HMP reduced overall bioaccessibility of β‐carotene from 32.1±6.2% to 24.1±5.7% (*p*<0.001). All other parameters also had a significant impact on the bioaccessibility of β‐carotene, that is, bile/pancreatin concentration (*p*<0.001), water bath shaking speed (*p*<0.001), and glass beads (*p* = 0.001). Surface tension, viscosity, and micelle size were less strongly affected by HMP addition (*p*<0.05), though not triglyceride lipolysis. The inhibitory effect of HMP varied depending on bile/pancreatin concentration and shear‐forces, with strongest reductions when β‐carotene bioaccessibility was highest at onset.

## Graphical abstract

High methoxyl pectin (HMP) significantly reduced β‐carotene bioaccessibility in vitro. This was observed at various enzyme/bile concentrations and shear forces.

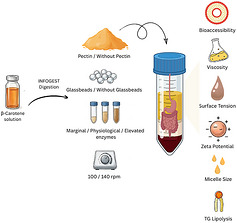

**Abbreviations**
AMD: Age‐related macular degeneration
DF: Dietary fiber
DM: Degree of methylesterification
FFA: Free fatty acid
GI: Gastrointestinal
HMP: High methoxyl pectin
LMP: Low methoxyl pectin
SD: Standard deviation
SGF: Simulated gastric fluids
SIF: Simulated intestinal fluids
TG: Triglycerides

## 1 Introduction

Carotenoids are naturally occurring lipophilic phytochemicals synthesized by a number of microorganisms, plants, fungi, and a few arthropods [1]. While they confer photo‐protection and light harvesting functions in plants [2], their human dietary intake has been associated with decreased levels of oxidative stress [3] reduced risk of age‐related macular degeneration (AMD) [4], and lowered risk of developing other non‐communicable diseases such as type‐2 diabetes [5] and some types of cancer [6]. Furthermore, some carotenoids such as β‐carotene and β‐cryptoxanthin are provitamin A compounds that contribute to vitamin A intake, particularly in vegetarian diets or in regions with limited access to preformed vitamin A from animal‐derived foods, such as parts of Africa and Asia [7].

Despite their ubiquitous distribution in fruits and vegetables, the bioavailability of carotenoids, that is, the fraction of a compound that can be used for its physiological function and/or stored, is typically low (5%–40%), owing, among other to their hydrophobicity [8]. The first bottleneck determining bioavailability involves carotenoid release from the food matrix and ultimately their incorporation within mixed micelles, that is, bioaccessibility.

Carotenoid micellization is strongly impacted by factors that affect the digestion of lipids, with lipids having in general, a positive impact on carotenoid bioavailability [9, 10]. Other factors enhancing bioaccessibility include well‐digestible proteins [11], while high amounts of divalent ions could hamper it [12, 13]. In addition, the amount and type of dietary fiber (DF) in the diet appears to impact carotenoid bioaccessibility [14].

Such a negative effect could be due to inhibition of lipase activity [15], bile acid binding and/or precipitation that limits emulsification capacity [16], or altered physicochemical properties of the chyme, e.g., increased viscosity [17]. All these effects can hinder micelle formation, promoting physical retention of carotenoids, either within structural assemblies of the food matrix (e.g., within plant cells) or through limiting lipid droplet processing. The increased chyme viscosity can also hinder the diffusion of enzymes to their substrates, of bile salts to unmicellized lipid constituents, and micellized carotenoids to the gut wall [18].

Several types of DF could reduce absorption of carotenoids. In a human study, supplementing 10 g of guar, alginate, cellulose, or wheat bran to a test meal decreased postprandial plasma response of β‐carotene, lutein, and lycopene by 30%–70%, depending on DF and carotenoid species [19]. Insoluble DF (e.g., cellulose and wheat bran) produced less negative effects than soluble DF. Another human study showed that supplementing a meal with citrus pectin strongly decreased the bioavailability of β‐carotene in plasma [20].

Pectin is a soluble DF found in many plants, mainly in citrus fruits and apples [21]. It is a linear polysaccharide composed of approximately 300–1000 monosaccharide units, predominantly d‐galacturonic acid, connected by α‐(1→4)‐glycosidic bonds. The functional properties of pectin are related to its chemical structure, including its degree of methyl‐esterification (DM), galacturonic acid (GalA) content, source and molecular weight [22, 23]. Pectins can be classified into “high‐methoxyl” (DM> 50%) or “low‐methoxyl” (DM< 50%) pectins, depending on the degree of methylation [24]. Typically, HMP forms gels under acidic conditions (pH < 3.5) when sufficient sugar (around 65% w/w) or other co‐solutes are present. In contrast, LMP forms gels primarily via ionic crosslinking with divalent cations and can gelate over a wider pH range [25]. It is thus commonly used in low‐calorie or dietetic products such as low fat yogurts [26, 27], while HMP is rather employed in jams, syrups and other processed foods [28].

In the current study, we aimed to investigate whether a potential negative effect of a commonly consumed soluble fiber, that is, HMP, on the bioaccessibility of β‐carotene, could be observed under a range of digestive conditions. For instance, it was deemed plausible that concentrations of digestive enzymes or strong shear forces, which may be compromised in certain diseases such as pancreatitis [29] or during aging [30] could impact carotenoid integration into mixed micelles, and that pectin may have more pronounced effects under such conditions. For this purpose, we employed the INFOGEST static consensus digestion model [31]. The measured endpoints included β‐carotene bioaccessibility, along with various physicochemical parameters of digesta such as viscosity and surface tension, as well as indicators of triglyceride lipolysis and micelle stability, including zeta‐potential and micelle size, all of which may be related to carotenoid bioaccessibility.

## 2 Materials and Methods

### 2.1 Chemicals and Enzymes

Pepsin from porcine gastric mucosa (≥250 U/mg, art. no. P7000), pancreatin from porcine pancreas (activity equivalent to 4× USP specifications, art. no. P1750) and porcine bile extract (art. no. B8631), as well as β‐carotene (≥ 97% all‐trans form, according to supplier and own HPLC analysis, Art. No. 22040) and HMP from apple (Art. No. 93854, degree of esterification 70%), that is, mostly methoxylation, as acetylation degree in apple is very low [32] were purchased from Sigma‐Aldrich (Overijse, Belgium). Peanut oil, typically free of native carotenoids (according to the USDA database (https://fdc.nal.usda.gov/) and own examination), was purchased from a local supermarket (Delhaize, Strassen, Luxembourg). Glass beads (diameter of 5 mm) were sourced from Merck Life Science BV/SRL (Overijse, Belgium).

Unless otherwise specified, all chemicals were of analytical grade or superior. Potassium chloride (≥99%), potassium dihydrogen phosphate (≥99%), sodium hydrogen carbonate (≥99%), sodium chloride (≥99.5%), magnesium chloride hexahydrate, ammonium carbonate, sodium hydroxide solution (1 M), and calcium chloride dihydrate (≥99%) were from Sigma‐Aldrich. n‐Hexane (≥95%), n‐heptane (≥99.3%), acetone (≥99%) and hydrochloric acid (1 M) were obtained from VWR (Leuven, Belgium).

### 2.2 Preparation of β‐Carotene, HMP and Enzyme Solutions

**FIGURE 1:**
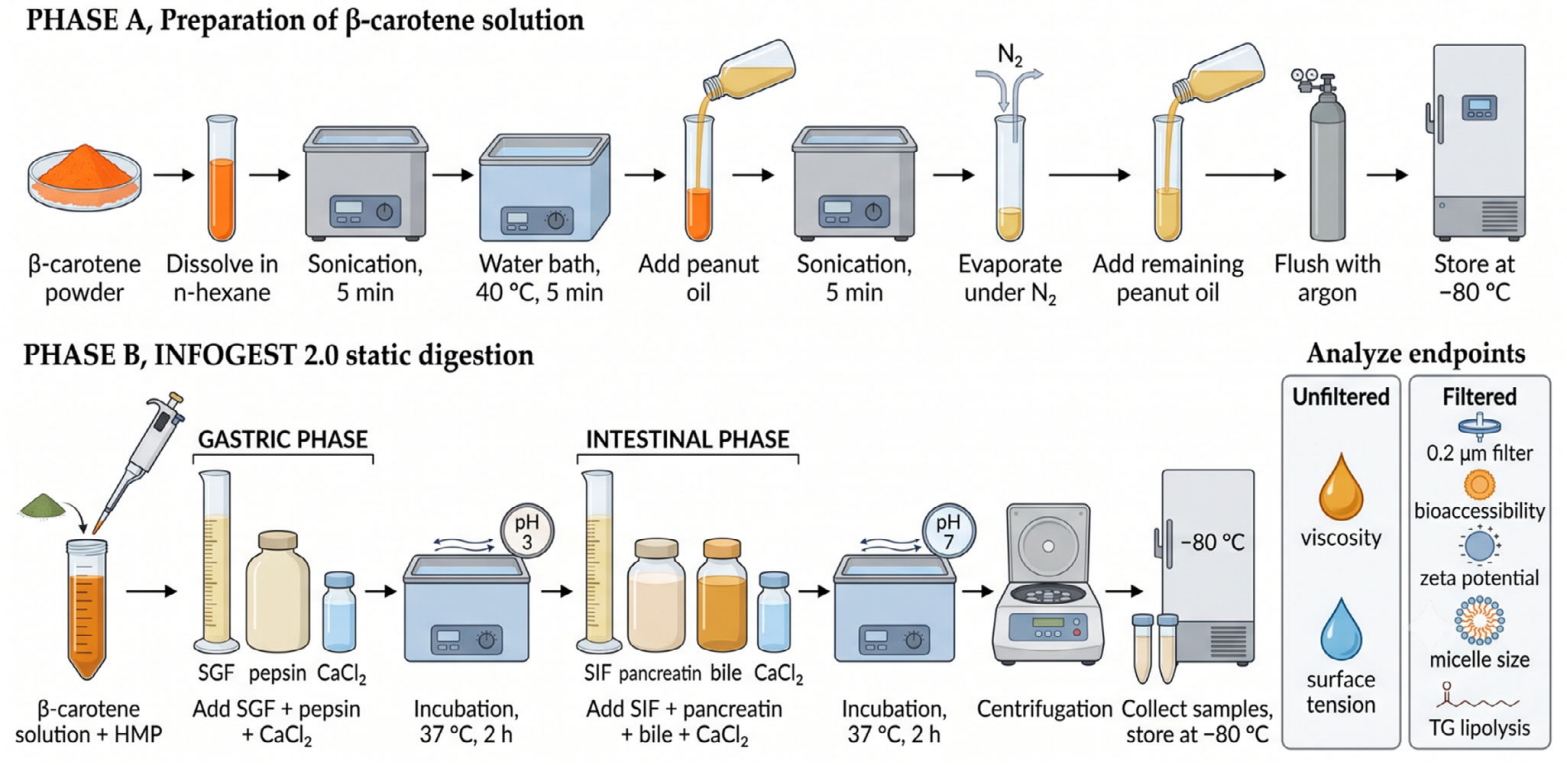
The figure demonstrates preparation of the β‐carotene solution and one representative INFOGEST run; in practice, digestions were performed under a range of conditions, with or without high methoxyl pectin (HMP), with or without glass beads, and at marginal, physiological, or elevated enzyme levels. To prepare the substrate, β‐carotene was dissolved in *n*‐hexane, mixed with peanut oil, dried under a stream of nitrogen, and stored at −80°C. Each digestion then passed through a gastric phase (pH 3) and an intestinal phase (pH 7) at 37°C. After centrifugation, viscosity and surface tension were measured directly in the unfiltered digesta, while bioaccessibility, zeta potential, micelle size, and triglyceride lipolysis were measured after passing the sample through a 0.2 µm nylon filter.

A β‐carotene standard solution was prepared by dissolving 2 mg of β‐carotene in 4 mL of n‐hexane. (Figure 1) This solution was then sonicated for 5 min and heated to 40°C for 5 min (Ultrasonic Cleaner, VWR Symphony, Massachusetts, USA). Thereafter, 2 mL of peanut oil was added, followed by further sonication and heating at 40°C for 5 min. *N*‐hexane was then removed by evaporation under a stream of nitrogen for ± 20 min. at 30°C (TurboVap LV from Biotage, Uppsala, Sweden). To this β‐carotene solution, another 2 mL of peanut oil was added to reach a final β‐carotene concentration of 0.5 mg/mL oil. This standard solution was flushed with argon gas, and stored at −80°C. A fresh solution of β‐carotene was prepared the day before the in vitro digestion experiments were carried out. The amount of β‐carotene added to each digesta was 75 µg (i.e., adding 150 µL of β‐carotene solution in oil), reflecting a high but achievable daily intake of 28 mg carotenoids per assumed 10 L of daily secreted digestive fluids [33], or 5–6 mg per 2–3 L of digestive fluid, simulating rather a single meal [34]. Similarly, the HMP concentration was standardized across all digesta at 30 mg per final volume of digesta (26 mL) to reach a concentration of 1.15 mg/mL, representing a bit less than 50% of the adequate intake (i.e., 25 g/d) of total dietary fibers for adults as defined by EFSA [35]. Practically, 30 mg of HMP was mixed with 6.35 mL of distilled water and solubilized before the addition of the 150 µL β‐carotene on a rotator mixer for ± 10 min. Simulated gastric fluids (SGF) and the simulated intestinal fluid (SIF) were prepared a day before the digestion and stored at room temperature as recommended by the INFOGEST model [31]. Pepsin solution was prepared in SGF to reach a final concentration of 2000 U/mL of the final gastric mixture.

Different pancreatin and bile concentrations were examined to understand their impact on lipid emulsification, though the ratio of pancreatin to bile was kept constant for three different conditions, which were supposed to reflect marginal, physiological, and elevated bile/pancreatin concentrations (Table 1), as reviewed earlier [36]. Based on the INFOGEST consensus digestion model [37], both bile and pancreatin were prepared in the same SIF solution. Pancreatin was weighed based on its trypsin activity (as certified by Sigma) to reach a final concentration of 100, 200 (employed as standard in our previous publication [36]), or 300 U/mL. The bile extract was weighed to reach a concentration of 3.4, 6.8, or 10.3 mg/mL in the final digestion volume (Table 1). Conditions with marginal pancreatin (100 U/mL) and bile (3.4 mg/ mL) that would still support β‐carotene micellization [38], but at the lower edge of what was reported as physiological [39], are denoted in the following as marginal digestive conditions. This potentially reflects conditions as in persons having exocrine pancreatic insufficiency [40]. In contrast, 200 U/mL pancreatin and 6.8 mg/mL bile are termed physiological, and 300 U/mL pancreatin and 10.3 mg/mL bile as elevated conditions. These elevated concentrations of bile/pancreatin reflected rather a higher physiological range [39], and were also evaluated to investigate whether they could override the presumably negative impact of HMP.

**TABLE 1:** Summary of digestion parameters studied as a function of HMP^**^ concentration.

Aspect of digestion	Conditions	Parameters chosen
**Matrix**	Amount of HMP (mg)	0, 30
	Glass beads (n) ^$^	0, 10
**Gastric Phase**	Shaking water speed (rpm) ^$^	100, 140
**Intestinal Phase**	Shaking water speed (rpm)	100, 140
	Bile (mg/mL)	3.4, 6.8*, 10.3
	Pancreatin (U/mL)	100, 200*, 300

All concentrations were per final volume of digesta at the respective digestive step. * Termed standard conditions, according to the European consensus digestion model [31], except rpm (rounds per minute) or glass beads which were not defined in the published model. ^$^Added/applied in order to simulate digestive shear forces. **High methoxyl pectin.

### 2.3 Simulation of Gastrointestinal (GI) Digestion

In vitro simulated gastrointestinal (GI) digestion was carried out using the harmonized INFOGEST 2.0 procedure, with digestive adjustments as described in Section 2.2. The oral digestion phase was omitted in this trial as the matrix was liquid, including no carbohydrates, and the study's primary focus was to zoom in on the interactions between DF and β‐carotene. In the gastric phase, 6.35 mL of HMP solution was combined with 150 µL of the β‐carotene solution in oil, similar as previously carried out [41]. Additionally, and also as these conditions were not precised in the original INFOGEST model, [31] the effectiveness of glass beads to simulate shear forces was evaluated by adding them into one set of samples. Next, 1 mL of pepsin solution was added to achieve enzymatic activities of 2000 U/mL in the final gastric volume. Subsequently, 33 µL of calcium dichloride (0.03 M) was introduced to reach a final concentration of 0.075 mM, and the pH was adjusted to 3 using 1 M hydrochloric acid. The total volume of each sample was then brought to 13 mL with the addition of distilled water. The samples were incubated in a shaking water bath (GFL 1083 from VEL, Leuven, Belgium) at 37°C for 2 h, with shaking speeds set at either 100 or 140 rpm to simulate various peristalses corresponding to a doubling of kinetic energy [36]. At the end of the gastric incubation, 7.8 mL of SIF, and 2.5 mL of pancreatin and bile extract solutions at various concentrations (100, 200, or 300 U/mL, based on trypsin activity for pancreatin, and 3.4, 6.8, or 10.3 mg/mL for the bile extract) were added to the chyme. Then, 260 µL of calcium dichloride (0.03 M) was added to reach a concentration of 0.3 mM in the final mixture. Before bringing the volume to a final volume of 26 mL, the pH was adjusted to 7.0 by the addition of sodium hydroxide solution (1 M). Then, the samples were incubated for 2 h at 37°C, with a shaking speed of 100 or 140 rpm.

### 2.4 Separation of β‐Carotene Bioaccessible Phase and Extraction

Following the completion of the intestinal phase of digestion, samples were centrifuged at 3200 × g for 1 h at 4 °C. From the unfiltered sample, 7.5 and 1.5 mL were aliquoted for viscosity and surface tension measurements, respectively. Additionally, 8 mL were collected from the middle aqueous phase and passed through a 0.2 µm nylon syringe filter. From the filtered fraction, 3 mL was used for β‐carotene bioaccessibility analysis (amount of β‐carotene in mixed micelles), while 1 mL aliquots were reserved for zeta potential, micelle size and free fatty acid determination. All aliquots were stored at −80 °C until further analysis. For the bioaccessibility analysis, 3 mL of the filtered aqueous phase was transferred into another 15 mL Falcon tube, followed by the addition of 6 mL of a n‐hexane:acetone mixture (2:1, v/v). The mixture was vortexed for 1 min and centrifuged at 3200 × g for 2 min. at 4 °C. The n‐hexane supernatant was collected into another Falcon tube. This extraction step was repeated twice using 4 mL of n‐hexane each time. All n‐hexane extracts were finally combined, evaporated to dryness under a nitrogen stream, flushed with argon, and stored at −80 °C until further analysis.

#### 2.4.1 Spectrophotometric Analysis

Spectrophotometric analysis of β‐carotene concentration in the micellar phase was carried out as described previously [42]. The dried residue was reconstituted in 750 µL of heptane and transferred to a 1 mL quartz cuvette (absorption cuvette, Hellma, New York, USA). The absorbance was measured between 300 and 700 nm (GENESYS 10S UV–Vis Spectrophotometer, Thermo Fisher Scientific, MA). As the digesta contained a single purified carotenoid (β‐carotene, ≥97% all‐trans) in peanut oil, which was free of native carotenoids (according to literature and own blank analyses), spectrophotometric determination was deemed appropriate. The concentration of β‐carotene was calculated by applying the Beer Lambert law, taking into account the molar absorption coefficient of β‐carotene in heptane (138,824 L mol^−1^ cm^−1^) [43] at peak absorption (450 nm). Finally, the percentage of β‐carotene retrieved from the micellar phase was used as a measure of bioaccessibility, and was expressed as the percentage of the solubilized amount of β‐carotene present in the aqueous phase of the filtered digesta after in vitro GI digestion, compared to the initial amount added to the sample before digestion.

$$\begin{array}{ccc} \text{Bioaccessibility}\;(\%) & = & \left(\beta \text{-carotene in filtered micellar phase}/\right.\\ & & \left.\beta \text{-carotene initially digested}\right)\times 100 \end{array}$$

### 2.5 Physicochemical Characterization of Digesta

#### 2.5.1 Viscosity and Shear Stress

The viscosity and shear stress behavior of the digesta was investigated using a double gap cylinder configuration in a MCR 302 WESP rheometer from Anton Paar (Graz, Austria). The frozen samples at ‐80°C were thawed shortly before the measurement and three biological replicates of each type were measured as followed: after temperature equilibration to 5°C, a pre‐shear phase with a shear rate of 5 s^−1^ was applied for 30 s. Then, viscosity and shear stress were determined as a function of shear rate between 0.1 and 131 s^−1^ and evaluated between 1 and 131 s^−1^, first in an increasing and then in a decreasing mode. The values of the increasing and decreasing modes were averaged as no hysteresis effects were observed. Finally, the curves of each sample type were averaged as well. Additionally, measurements were performed at a constant shear rate of 131 s^−1^ during the stabilization period of about 30 s between the rising and falling modes. The 50 measurements were statistically analyzed and compared with OriginPro 2019b from OriginLab Corporation (Northampton, MA, USA).

#### 2.5.2 Surface Tension

The surface tension of digesta, pre‐conditioned at 20 ± 0.1°C, was determined using the weight‐drop method as described by Gianino (2006) [44]. The air–water interfacial properties of the digesta were calculated using the following equation:

$$\sigma digesta=\frac{Mdigesta}{MH2\;O}\;\;\times \;\sigma H2O$$

with σH_2_O = 72.5 mN m^−1^ at 20°C, [44] σH2O being the surface tension of pure water, σ_digesta_ being the surface tension of digesta, M_digesta_ being the mass of one drop of digesta, and M_H2O_ being the mass of one drop of water.

#### 2.5.3 Micelle Size and Zeta Potential Analysis

Aliquots from the filtered bioaccessible fractions were used for analysis of the micelle size and ζ‐potential, and the measurements were done at room temperature with at least three biological replicates. The intensity‐weighted mean hydrodynamic diameter (Z‐average) and ζ‐potential were determined by dynamic light scattering and laser Doppler micro‐electrophoresis, respectively, by using a Zetasizer Nano Zs instrument (Malvern Instruments, Malvern, UK).

#### 2.5.4 Triglyceride Lipolysis

Quantification of free fatty acids was done following the GI digestion to estimate the extent of triglyceride lipolysis. This was accomplished by employing the Cayman Free Fatty Acid Fluorometric Assay kit and the protocol provided by the manufacturer (Cayman Chemical, art. no. 700310, Ann Arbor, MI).

### 2.6 Statistical Analyses and Data Visualization

In order to minimize day‐to‐day variations between experiments, bioaccessibility of β‐carotene was normalized to a daily standardized control sample that was assessed for each digestion. However, the use of two controls, one for 100, one for 140 rpm, was unavoidable.

Unless otherwise stated, all values are expressed as the mean ± standard deviation (i.e., estimated marginal means and pooled SD from linear mixed models). Four replicates were carried out for each individual digestive condition. Statistical analysis was performed using SPSS 22 software (SPSS Inc., Chicago, IL).

Normal distribution of data was verified by Q‐Q –plots and equality of variance by box plots and scatter plots of standardized residuals against standardized predicted values. Linear mixed models were developed, with the concentration of HMP (0 or 30 mg/mL), concentration of bile/pancreatin (3 levels), kinetic energy/shaking speed (100 and 140 rpm) and presence/absence of glass beads (i.e., two levels), labeled as fixed, independent factors and β‐carotene bioaccessibility as the observed dependent factor. In addition to main effects, all interactions were studied as well, but were removed stepwise when being non‐significant, that is, only significant interactions were kept in the final model. When needed and significant interactions were encountered, one of the interacting factors was kept constant and the model re‐run. For comparing individual groups, group‐wise comparisons were carried out. *P*‐values <0.05 (2‐sided) were considered statistically significant different. Furthermore, post hoc tests (Fischer's protected LSD tests for comparison of ≤3 groups) were conducted to avert any false positives following multiple comparisons. Correlation analyses using Pearson correlation coefficients between FFA release, zeta‐potential surface tension, and β‐carotene bioaccessibility were also conducted.

## 3 Results

### 3.1 Overall Effects on Bioaccessibility of β‐Carotene

**FIGURE 2:**
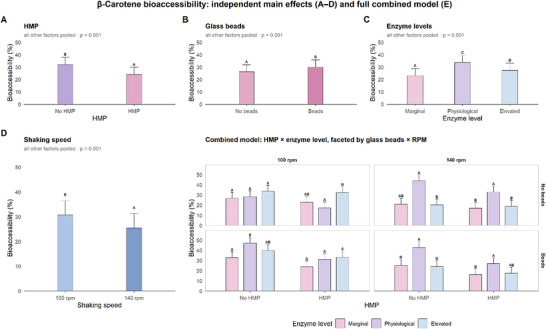
High methoxyl pectin reduces β‐carotene bioaccessibility across various gastrointestinal digestion conditions. Panels (A–D) present the independent effect of each factor on mean β‐carotene bioaccessibility (%), with all other factors statistically pooled: (A) high methoxyl pectin (HMP; 0 vs 30 mg per digesta), (B) glassbeads (0 vs 10 units), (C) bile/pancreatin concentration (marginal, physiological, elevated), and (D) water bath shaking speed (100 vs 140 rpm). Panel (E) presents the full combined model: the *x*‐axis groups data by HMP concentration (0 and 30 mg), columns represent agitation speed (100 rpm left, 140 rpm right), and rows represent amount of glass beads (0 units top, 10 units bottom). Within each panel of (E), bars are color‐coded by bile/pancreatin concentration: marginal (pink), physiological (purple), and elevated (blue). Bars represent estimated marginal means and error bars indicate pooled standard deviation, both derived from linear mixed models. In all panels, letters (A, B, C) indicate homogeneous subsets based on Tukey's post‐hoc test following the linear mixed model; within each panel, bars sharing the same letter are not significantly different (*p* > 0.05).

This study found that overall, that is, considering all digestive conditions pooled, HMP reduced the fractional bioaccessibility of β‐carotene by almost 25% compared to controls, from 32.1 ± 6.2% to 24.1 ± 5.7% (Figures 2, *p* < 0.001). The linear mixed model confirmed statistically significant effects of individual parameters (Table 2), including bile/pancreatin concentration (*p* < 0.001), glass beads (*p* = 0.001), HMP (*p* < 0.001), and rpm (*p* < 0.001). There were significant two‐way interactions for bile/pancreatin and HMP interactions (*p* = 0.004), bile/pancreatin and rpm interactions (*p* < 0.001), glass beads and rpm (*p* < 0.001) and glass beads and HMP (*p* = 0.029). Furthermore, a statistically significant triple interaction between glass beads, bile/pancreatin and rpm was also observed (*p* = 0.006). Other interactions, including the four‐way interaction between rpm, glass beads, bile/pancreatin, and HMP, on β‐carotene bioaccessibility were not significant.

**TABLE 2:** Estimated marginal means (± SD) of β‐carotene bioaccessibility under varying levels of HMP^*^, bile/pancreatin concentration, shaking speed (RPM), and presence of glass beads in an in vitro digestion model.

Factor	Levels	Estimated Marginal Mean ± SD	p‐value
HMP (mg)	0	32.1 ± 6.2	
	30	24.1 ± 5.7	*p* <0.001
Glassbeads (units)	0	26.1 ± 6.2	
	10	30.0 ± 5.8	*p* = 0.001
Bile/Pancreatin concentration	Marginal (3.4 mg/mL bile + 100 U/mL pancreatin)	23.1 ± 6.4	
	Physiological (6.8 mg/mL bile + 200 U/mL pancreatin)	33.7 ± 5.8	
	Elevated (10.3 mg/mL bile + 300 U/mL pancreatin)	27.4 ± 5.8	*p*<0.001
Rpm	100	30.7 ± 5.9	
	140	25.4 ± 5.9	*p*<0.001

*High methoxyl pectin.

### 3.2 Effect of Glass beads on β‐Carotene Bioaccessibility

Overall, the incorporation of glass beads significantly increased the relative mean bioaccessibility of β‐carotene compared to controls, from 26.1 ± 6.2% to 30.0 ± 5.8% (*p* = 0.001). As a significant interaction between glass beads and HMP was observed (see 3.1), we studied the effect of glass beads with/without HMP in separate models (Figure  2). In the absence of glass beads (irrespective of the bile/pancreatin concentration or rpm level), addition of HMP resulted in a reduction of mean bioaccessibility from 29.0 ± 6.2% with no HMP to 23.4 ± 5.5% (19% reduction, *p* < 0.001). A similar trend was observed in case of samples with glass beads, where adding HMP caused a significant (*p* < 0.001) reduction of bioaccessibility from 35.2 ± 6.0% for 0 mg HMP to 24.7 ± 6.0% (30% reduction) for 30 mg HMP.

As the interaction between glass beads and rpm was also significant, further individual linear models were run. It was observed that the samples with glass beads (with statistically pooled enzyme and bile/pancreatin concentrations) had a higher mean bioaccessibility at 100 rpm than at 140 rpm (34.6 ± 5.8% vs 25.3 ± 5.8%, *p* < 0.001). Without glassbead addition, the bioaccessibility at 100 rpm vs. 140 rpm was not significantly different.

### 3.3 Effect of Bile and Pancreatin Concentration on β‐Carotene Bioaccessibility

A significant effect of bile/pancreatin concentration was observed on the overall bioaccessibility of β‐carotene (all the other factors pooled, see 3.1). Interestingly, the mean bioaccessibility was highest for physiological bile/pancreatin concentrations (200 U/ml pancreatin and 6.8 mg/mL bile), that is, 33.7 ± 5.8%, while it decreased with elevated bile/pancreatin concentrations (300 U/mL pancreatin and 10.3 mg/mL bile) to 27.4 ± 5.8%, and was lowest for marginal bile/pancreatin concentrations (100 U/mL pancreatin and 3.4 mg/mL bile), that is, 23.1 ± 6.4%. Pairwise comparisons showed that the physiological concentration gave rise to significantly different bioaccessibility from both the elevated and marginal bile/pancreatin concentrations (*p* < 0.001). Additionally, the marginal bile/pancreatin concentration differed significantly from the high concentration (*p* = 0.002).

As a significant interaction between bile/pancreatin concentrations and HMP was observed (*p* = 0.004), further individual linear mixed models were run, with bile/pancreatin concentrations kept constant. It was observed that the presence or absence of HMP modulated the influence of bile/pancreatin concentration on the bioaccessibility of β‐carotene; that is, at elevated bile/pancreatin concentration, the mean bioaccessibility dropped significantly (*p* = 0.027) from 29.4 ± 4.8% for samples without HMP to 25.4 ± 4.8% (13.6%) for samples with HMP. Similarly, for physiological bile/pancreatin concentration, the mean β‐carotene bioaccessibility was reduced from 40.4 ± 6.2% in samples without HMP to 27.0 ± 6.2% (or 33.2%) in samples with added HMP (*p* < 0.001). Furthermore, at marginal bile/pancreatin concentrations, the mean bioaccessibility decreased from 25.8 ± 6.7% in samples without HMP to 19.8 ± 6.0% (23.3%) in samples with added HMP (*p* = 0.003). These differences between 0 and 30 mg HMP at each bile/pancreatin concentration were all statistically significant from one another.

Another factor that influenced the effect of bile/pancreatin concentrations on the bioaccessibility of β‐carotene was shaking speed of the water bath, as suggested by the significant interaction between bile/pancreatin concentrations and rpm (Section 3.1). At elevated bile/pancreatin concentrations, increasing the rpm from 100 to 140 rpm significantly reduced mean β‐carotene bioaccessibility, from 34.7 ± 4.8% to 20.1 ± 4.8% (*p* < 0.001). Similarly, the mean bioaccessibility at marginal bile/pancreatin concentrations dropped from 26.3 ± 6.7% at 100 rpm to 19.3 ± 6.7% at 140 rpm (*p* < 0.001). Somewhat surprisingly, it was only at physiological bile/pancreatin concentrations that the mean bioaccessibility was increased (*p* = 0.013) upon increasing the rpm, that is, from 30.9 ± 6.2% at 100 rpm to 36.5 ± 6.2% at 140 rpm.

### 3.4 Effect of Shaking Speed of Water Bath on β‐Carotene Bioaccessibility

Overall, the rpm levels (speed of the water bath) reduced bioaccessibility significantly (Section 1, 3), from 30.7 ± 5.9% at 100 rpm to 25.4 ± 5.9% at 140 rpm. In addition to the two‐way interactions of rpm and glass beads and rpm and bile/pancreatin (Section 3.1), another significant triple interaction was observed between glass beads, bile/pancreatin concentration, and rpm (Section 3.1). The recorded mean bioaccessibility for the no‐glass bead group was highest at physiological bile/pancreatin concentrations at 140 rpm (38.3 ± 4.3%) and lowest at marginal levels under the same rpm condition (18.7 ± 7.3%). In the presence of glass beads, the highest bioaccessibility, that is, 39.0 ± 7.2%, was recorded at physiological bile/pancreatin concentration at 100 rpm, while the lowest value, that is, 20.5 ± 5.5%, was found at marginal bile/pancreatin concentration at 140 rpm.

### 3.5 Effect of Factors Influencing the Physicochemical Properties of the Digesta on β‐Carotene Bioaccessibility

#### 3.5.1 Viscosity and Shear Stress

Irrespective of bile/pancreatin concentration and glass beads, the samples showed Newtonian behavior above shear rates of 10 s^−1^. HMP, however, was the only factor that affected the viscosity of samples significantly (*p* = 0.002), that is, a low but marked difference between samples with vs. without HMP. Consequently, samples with HMP showed slightly higher shear stress values in comparison to the sample without HMP. The statistical analysis of the viscosities at a shear rate of 131 s^−1^ supported this finding. The mean values for the viscosities of the investigated samples were only slightly higher than water, and the resulting maxima of the normal distributions were found at 1.77 mPa.s for samples without and 2.44 mPa.s for the samples with HMP.

#### 3.5.2 Surface Tension

The primary factors found to significantly influence surface tension were HMP (*p* < 0.001), bile/pancreatin concentration (*p* < 0.001), and rpm (*p* = 0.016). Additionally, significant two‐way interactions were observed between glass beads and bile/pancreatin concentration (*p* = 0.013), as well as between HMP and bile/pancreatin concentration (*p* < 0.001). Notably, significant three‐way interactions between glass beads, HMP, and bile/pancreatin concentrations (*p* = 0.010), were also observed. The analysis showed that increasing HMP from 0 to 30 mg led to a small but significant reduction in surface tension from 25.7 ± 1.7 mNm^−1^ to 24.4 ± 1.6 mNm^−1^. The marginal bile/pancreatin concentrations exhibited the highest surface tension values at 26.6 ± 1.4 mNm^−1^, followed by elevated bile/pancreatin concentrations at 24.6 ± 1.0 mNm^−1^, which were slightly higher compared to physiological bile/pancreatin concentrations at 23.8 ± 1.0 mNm^−1^. When rpm was raised from 100 to 140, the surface tension decreased from 25.4 ± 1.6 mNm^−1^ to 24.6 ± 1.6 mNm^−1^. However, all these changes were considered to be of low physiological significance, and hence further interactions were not studied to avoid biological over‐interpretation.

#### 3.5.3 Triglyceride Lipolysis

In case of TG lipolysis, bile/pancreatin concentrations (*p* < 0.001) and glass beads (*p* = 0.027) significantly impacted triglyceride lipolysis, the impact of HMP was borderline significant (*p* = 0.056), with higher amounts of HMP tending to result in higher lipolysis. Moreover, significant two‐way interactions between bile/pancreatin concentrations and glass beads (*p* < 0.001), bile/pancreatin concentrations and rpm (*p* < 0.001), bile/pancreatin concentrations and HMP (*p* = 0.021) and glass beads and rpm (*p* = 0.004) were also observed. Note that 100% would equal complete lipolysis of triglycerides into monoglycerides and fatty acids. Elevated bile/pancreatin concentrations resulted in the highest triglyceride lipolysis at 124.7 ± 37.9%, which was significantly greater than both marginal (*p* = 0.006) and physiological levels (*p* < 0.001). Marginal bile/pancreatin concentrations showed intermittent lipolysis at 102.3 ± 29.3%, significantly higher than physiological levels at 79.5 ± 37.9% (*p* = 0.005). Samples containing glass beads showed higher lipolysis of 110.0 ± 26.8 % compared to those without, 94.4 ± 45.9%.

#### 3.5.4 Micelle Size

The size measurements revealed two distinct populations. One for marginal and physiological bile/pancreatin concentrations (with rpm, glass beads and HMP pooled) with average hydrodynamic diameters above 100 nm and thus not considered mixed micelles. Another population was observed only for elevated bile/pancreatin concentrations (pooled rpm, glass beads, and HMP), with measures below 10 nm. It was thus decided to only consider the true micelles (size below 10 nm), observed only for elevated enzyme bile/pancreatin concentrations at 100 and 140 rpm for further analysis. The findings revealed a small but statistically significant impact of HMP on micelle size, with higher amounts of HMP producing larger mixed micelle sizes, that is, 6.1 ± 0.4 nm vs. 5.8 ± 0.4 nm (*p* = 0.008). Glass beads and rpm did not have a significant effect. Regarding interactions, the two‐way interaction between glass beads and rpm was significant (*p* = 0.019). While no significant effects were found for no glass beads regarding HMP and rpm, with the presence of glass beads, HMP (though not rpm) had a significant effect on micelle size (*p* = 0.029), with larger mixed micelle size in the presence of HMP, that is, 6.2 ± 0.6 nm vs. 5.7 ± 0.4 nm for the group without HMP.

#### 3.5.5 Zeta Potential

**FIGURE 3:**
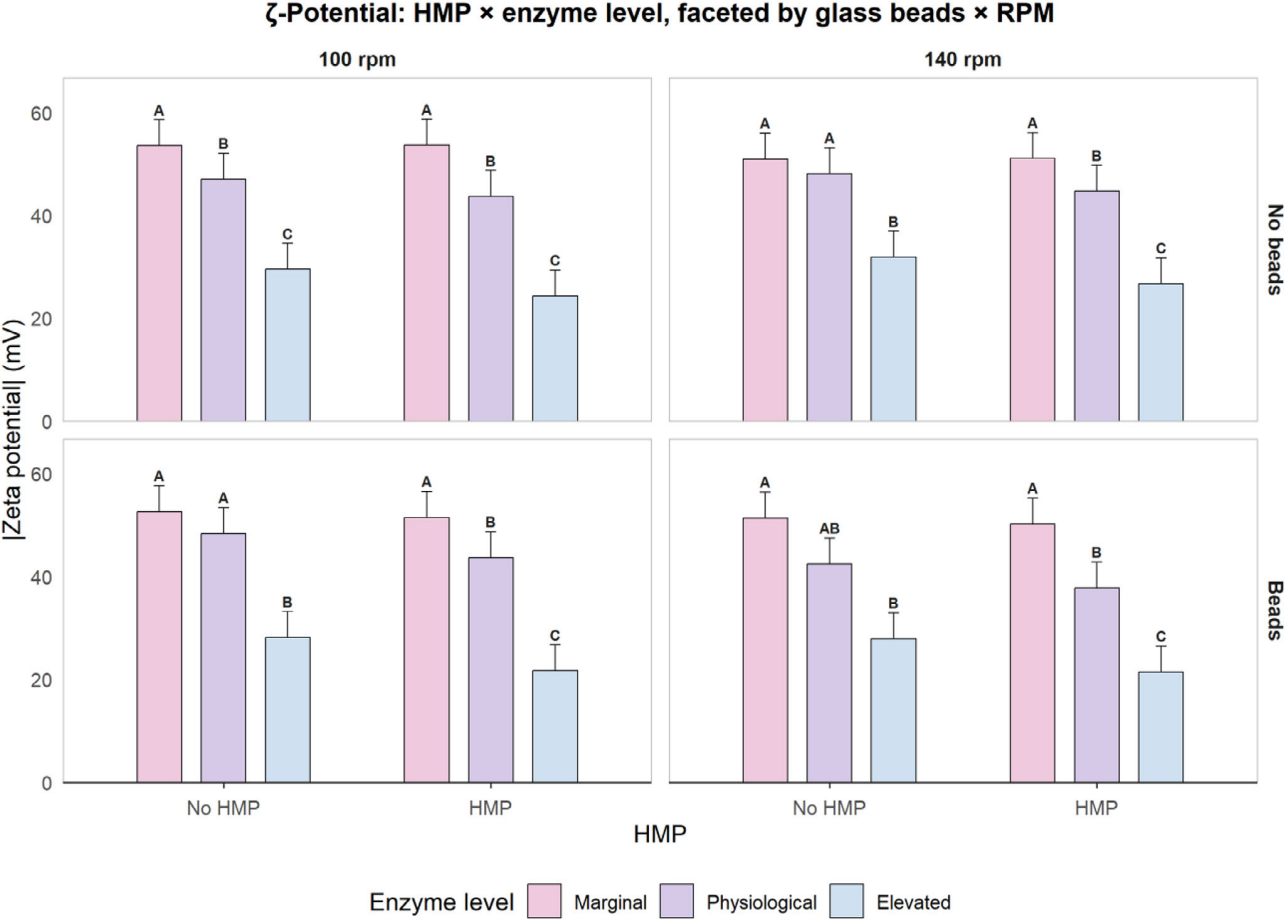
The figure displays mean absolute zeta potential values in millivolts (mV) on the *y*‐axis across different digestive conditions. Bars represent estimated marginal means and error bars indicate pooled standard deviation, both derived from linear mixed models. The *x*‐axis represents the amount of high methoxyl pectin (HMP) per digesta (0 and 30 mg). The figure is divided into four panels based on water bath shaking speed (100 and 140 rpm) and amount of glass beads (0 and 10 units). Within each panel, three bars are shown for each HMP level, corresponding to bile/pancreatin levels: marginal (pink), physiological (purple), and elevated (blue). Zeta potential values ranged from approximately 20 to 60 mV. Homogeneous subset groups following the linear mixed model, determined by Tukey's post‐hoc test, are indicated above each bar using letters A, B, and C; bars sharing the same letter are not significantly different from each other (*p* > 0.05).

All zeta‐potential values were negative, and thus absolute values are reported (Figure 3). Zeta potential was significantly affected by glass beads (*p* = 0.029), bile/pancreatin concentration (*p*<0.001) and HMP (*p* = 0.001). Pooling other factors, the addition of glass beads reduced the zeta potential compared to the samples without glass beads from 42.0 ± 5.5 to 39.8 ± 5.0 mV. In case of bile/pancreatin concentrations, the samples with marginal bile/pancreatin concentration had the highest zeta potential at 51.7 ± 5.6 mV followed by physiological bile/pancreatin concentration at 44.5 ± 5.0 mV, with lowest zeta potential observed at elevated bile/pancreatin concentrations at 26.5 ± 5.0 mV (considering other factors pooled). Each bile/pancreatin concentration was significantly different from one other (*p* < 0.001). HMP supplementation to the samples led to a decrease in absolute zeta potential values compared to controls, from 42.6 ± 5.5 to 39.2 ± 5.0 mV (all other factors pooled).

#### 3.5.6 Correlation Analysis

**FIGURE 4:**
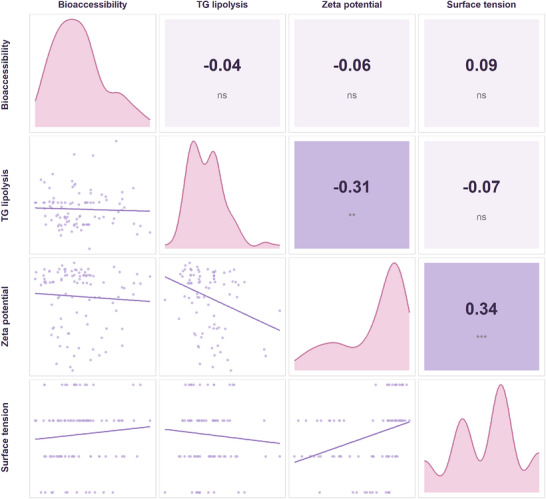
Pairwise relationships among four digestion endpoints measured in the bioaccessible fraction (*n* = 96): β‐carotene bioaccessibility, triglyceride lipolysis (free fatty acid release), absolute zeta potential, and surface tension. The lower triangle displays scatter plots with linear fit lines for each pair of variables; the diagonal shows the distribution (kernel density) of each individual variable; and the upper triangle reports the corresponding Pearson correlation coefficient (r) with its significance level (ns = not significant), with cells shaded to highlight stronger correlations (|r| ≥ 0.30). Triglyceride lipolysis was significantly negatively correlated with absolute zeta potential (r = −0.31, *p* = 0.002), and surface tension was significantly positively correlated with absolute zeta potential (r = 0.34, *p* = 0.001). β‐Carotene bioaccessibility did not show a significant correlation with any individual physicochemical endpoint.

Pearson correlation analysis was conducted to explore associations between bioaccessibility, triglyceride lipolysis, surface tension, and zeta potential (Figure 4). Triglyceride lipolysis was inversely correlated with absolute zeta potential (r = –0.31, *p* = 0.002) and surface tension was positively correlated with absolute zeta potential (r = 0.34, *p* = 0.001).

When investigating the correlation between the bioaccessibility of β‐carotene without HMP addition and the fractional (per cent) reduction of bioaccessibility with HMP, a significant correlation was observed, that is, a higher starting bioaccessibility without HMP was associated with a stronger reduction of bioaccessibility following HMP addition (*p* < 0.001, r = 0.775).

## 4 Discussion

This study demonstrates that the adverse effect of physiologically relevant levels of HMP, at amounts that are plausibly and frequently consumed and that are widely applied in the food industry, on β‐carotene bioaccessibility, persisted across a range of digestive conditions. This encompassed varying pancreatin and bile concentration, and peristalsis, which may be relevant for elderly or in case of diseases impacting enzyme secretion such as pancreatitis. Across all conditions, HMP (on average) reduced β‐carotene bioaccessibility by approx. 25% vs HMP‐free controls.

This inhibitory impact is consistent with previous findings in vivo and in vitro. For example, Riedl et al. (1999) [19] demonstrated that pectin supplementation to a meal (unspecified degree of methylation) reduced the bioavailability of lutein, lycopene, and β‐carotene in humans by up to 40%. The strong negative impact of HMP on β‐carotene bioaccessibility could be attributed to the gel‐forming nature of HMP. In theory, pectin gels may (a) bind divalent ions, essential cofactors for lipase activity [45, 46]; (b) entrap bile salts within its matrix, reducing their ability to emulsify dietary lipids [47]; (c) alter the physical structure and stability of emulsions by binding to lipid droplets, inhibiting the formation of mixed micelles, which are critical for the solubilization and transport of carotenoids during digestion [48]; (d) increase the viscosity of the chyme, hindering the mobility and interaction of digestive enzymes with their substrates, potentially slowing down the digestion process and reducing the efficiency of micellization [49].

In the present study HMP sourced from apples was used. Most natural food sources are typically rich in HMP, but this type is also frequently employed by the food industry in processed foods as a gelling agent such as in jams and jellies [50]. HMP forms gels under acidic conditions, e.g., at pH ≤ 3.5 (such as present in the gastric phase of digestion), as well as the presence of soluble solids, mainly through hydrogen bonding [51]. Contrarily, LMP gels can form over a broader pH range (3–7) in the presence of divalent cations such as Ca^2^^+^ (conditions that may be present in the small intestine following meal ingestion), to create gels by ionic cross‐linking between the negatively charged free carboxyl groups. A previous in vitro gastrointestinal digestion study showed that the rate and extent of lipid digestion decreased with increasing pectin molecular weight and methoxylation, with FFA release after 120 min being 47%, 70%, and 91% (w/w) for HMP, medium methoxylated pectin, and LMP, respectively [52]. The HMP also showed higher viscosity and lower stability (zeta potential) of the emulsion against coagulation vs. LMP. This is in line with the overall negative effect of HMP on β‐carotene bioaccessibility observed in the present study. We can only speculate that LMPs may have a less negative impact on β‐carotene bioaccessibility compared to HMPs, as demonstrated earlier [53].

Despite the general negative impact of HMP on β‐carotene bioaccessibility, the fractional (i.e., relative percentage) reduction of bioaccessibility in the presence of HMP was stronger for physiological bile/pancreatin concentrations, that is, when bioaccessibility (without HMP) was higher, than at elevated concentrations, followed by marginal bile/pancreatin concentrations. This tendency was observed for all conditions, that is, in the absence/presence of glass beads or lower/higher rpm, and was supported by a correlation between higher bioaccessibility at onset (without HMP) and a stronger negative percentage effect on bioaccessibility with the addition of HMP. Thus, by adding HMP, differences in β‐carotene bioaccessibility across conditions were leveled out. The findings that marginal digestive conditions with lower bile/pancreatin concentration resulted in lower β‐carotene bioaccessibility was observed also in a previous study [36], while it is more difficult to explain the low bioaccessibility observed at elevated bile/pancreatin concentrations. As zeta potential was also lowest at elevated concentrations of bile/pancreatin, it appeared that repulsive forces and stability of mixed micelles in emulsions were compromised under these conditions. It has been proposed that a too high emulsifier concentration can destabilize emulsions, reduce interfacial film strength, or even produce a salting‐out effect of the emulsifier [54]. We could not clearly support this with any correlations with mixed micelle size measures across digestive conditions, likely due to the presence of too high concentrations of larger particles, presumably lipid droplets, present in the digesta, especially at lower concentrations of bile/pancreatin, impeding particle size measures.

A factor clearly impacted by the presence of HMP was viscosity, which was higher in the presence of HMP, due to its gel‐forming nature [55]. A higher HMP level also reduced surface tension, suggesting that it could act as a mild surfactant, which may enhance the formation and stability of emulsions [56, 57]. Surface‐active components can reduce interfacial tension, thereby enhancing dispersion [58]. However, changes in surface tension (approx. 5%) were considered small and likely not biologically relevant. Higher viscosity during digestion on the other hand was shown to hinder enzyme‐substrate interactions, potentially slowing down lipid digestion [59]. However, no decreased triglyceride lipolysis with the addition of HMP was observed in the present study, and perturbed enzymatic activity is thus unlikely to explain the reduction in bioaccessibility. It is possible that yet higher amounts of HMP could produce a negative effect, as another study with threefold higher concentrations of HMP demonstrated a measurable impact on FFA release [14]. However, increased viscosity may also hamper directly carotenoid incorporation into mixed micelles [60] or, alternatively, viscosity may not have been causally implicated in the reduction of β‐carotene micellization.

Addition of HMP also reduced absolute zeta potential, by approximately 8% on average. A higher net charge of the particles, due to electrostatic repulsion, would rather stabilize micelles [61]. Hence an observed decrease would rather indicate a reduced stability of micelles [62], in line with the observed reduced β‐carotene bioaccessibility.

A novel aspect of this study was incorporating glass beads during digestion to better simulate shear forces during digestion, which is not mentioned in the INFOGEST model [31]. Shear‐forces are important for disintegration and emulsification of food matrices [63], known to reduce particle size, potentially increasing surface area of the substrate and enhancing lipase accessibility to lipid droplets, producing smaller ones [64]. While a significant increase with glass beads on triglyceride lipolysis was observed, cleavage appeared fairly complete with or without glass beads. However, adding glass beads also improved significantly β‐carotene bioaccessibility, at least at lower rpm (100), though not at higher (140) rpm. However, glass beads were unable to override the negative impact of HMP on β‐carotene bioaccessibility. Rather, when adding HMP to the digestion containing glass beads, its negative impact on bioaccessibility was more (though not significantly) pronounced (relative reduction of 30% vs. 19% without glass beads), so that with or without glass beads, the final bioaccessibility of β‐carotene (with HMP) was similar.

In contrast to adding glass beads, increasing the rpm of the waterbath, also investigated as a means to simulate higher peristalsis, generally reduced β‐carotene bioaccessibility, despite not negatively impacting triglyceride lipolysis, zeta potential, or micelle size, suggesting that other factors were responsible for the lower general micellization at 140 rpm [65]. The present findings also differ from an earlier study when increasing rpm from 75 to 100 rpm generally improved β‐carotene bioaccessibility [36], perhaps suggesting an optimum rpm for fostering bioaccessibility. The results also suggest that increasing rpm simulates different aspects of peristalsis compared to the addition of glass beads. More studies on the neglected effects of peristalsis in in vitro models are warranted.

## 5 Conclusions

The present study highlights the adverse effect of HMP, a commonly encountered soluble dietary fiber, also used as an additive in many processed foods, on β‐carotene bioaccessibility. The negative effect prevailed under various digestive conditions, with an average reduction of ∼25%. The negative effects of HMP on β‐carotene bioaccessibility were more pronounced when the baseline bioaccessibility (without HMP) was high. At low baseline values, the relative reduction appeared less evident. Thus, in individuals with digestive disorders, such as pancreatitis, where enzyme activity and digestive efficacy are impaired, such further reductions from an already low bioaccessibility caused by HMP may be difficult to detect. The negative impact of HMP on β‐carotene bioaccessibility is likely due to HMP's gel‐forming properties, and, as observed, increasing viscosity and limiting stability of the micelle fraction as reflected by lower absolute zeta potential, while triglyceride lipolysis was not negatively impacted. While further research is necessary to fully elucidate the interactions between HMP and β‐carotene bioaccessibility, this study provides insights into the negative effects of HMP at different enzyme/bile concentrations and simulated peristalsis, potentially relevant for parts of the population such as those with impaired digestion (Supporting Information).

## Conflicts of Interest

The authors declare no conflicts of interest.

## Supporting information




**Supporting File**: mnfr70562‐sup‐0001‐SuppMat.docx.

## Data Availability

The data that support the findings of this study are available on request from the corresponding author. The data are not publicly available due to privacy or ethical restrictions.
