# Supplementary material for: High Methoxyl Pectin Consistently Reduces β‐Carotene Bioaccessibility Across Various Gastrointestinal Digestion Conditions
Source: Mol Nutr Food Res. 2026 Aug 5;70(15):e70562. doi: 10.1002/mnfr.70562 (PMC13439340; doi:10.1002/mnfr.70562)
Supplement: Supplementary file 1 [file MNFR-70-e70562-s001.docx]

**Supplementary Table 1**- Mean bioaccessibility (%) ± SD for β-carotene from 4 replicates under different enzyme levels, glass bead addition, RPM, and pectin conditions. Given are estimated marginal means and pooled standard deviations as obtained from linear mixed models.

| **Pectin (mg)** | **Glassbeads (numbers)** | **Bile/pancreatin*** | **RPM **** | **Mean bioaccessibility (%)** | **SD** |
| --- | --- | --- | --- | --- | --- |
| 0 | 0 | Marginal | 100 | 26.2 | 5.3 |
| 0 | 0 | Marginal | 140 | 21.1 | 8.2 |
| 0 | 0 | Physiological | 100 | 27.8 | 4.7 |
| 0 | 0 | Physiological | 140 | 43.3 | 2.3 |
| 0 | 0 | Elevated | 100 | 34.5 | 7.4 |
| 0 | 0 | Elevated | 140 | 20.9 | 3.6 |
| 0 | 10 | Marginal | 100 | 32.5 | 10.4 |
| 0 | 10 | Marginal | 140 | 26.1 | 1.1 |
| 0 | 10 | Physiological | 100 | 50.4 | 2.7 |
| 0 | 10 | Physiological | 140 | 40.2 | 6.0 |
| 0 | 10 | Elevated | 100 | 42.5 | 5.1 |
| 0 | 10 | Elevated | 140 | 19.7 | 2.4 |
| 30 | 0 | Marginal | 100 | 24.0 | 3.4 |
| 30 | 0 | Marginal | 140 | 16.3 | 3.8 |
| 30 | 0 | Physiological | 100 | 17.7 | 3.4 |
| 30 | 0 | Physiological | 140 | 33.2 | 6.1 |
| 30 | 0 | Elevated | 100 | 31.1 | 5.1 |
| 30 | 0 | Elevated | 140 | 18.0 | 1.7 |
| 30 | 10 | Marginal | 100 | 24.0 | 3.0 |
| 30 | 10 | Marginal | 140 | 14.9 | 1.9 |
| 30 | 10 | Physiological | 100 | 27.6 | 7.4 |
| 30 | 10 | Physiological | 140 | 29.4 | 10.5 |
| 30 | 10 | Elevated | 100 | 30.7 | 6.1 |
| 30 | 10 | Elevated | 140 | 21.7 | 2.4 |

* Bile/pancreatin ratio:

- “Marginal” refers to the lowest tested bile (3.4 mg/mL) and pancreatin (100 U/mL) concentrations.
- “Physiological” corresponds to medium bile (6.8 mg/mL) and pancreatin (200 U/mL) concentrations.
- “Elevated” indicates the highest tested bile (10.3 mg/mL) and pancreatin (300 U/mL) concentrations.

** Revolutions per minute of shaking water bath.

**Supplementary Table 2-** Individual β-carotene bioaccessibility (%) and absolute concentration (µg) recovered in the filtered micellar phase for every digestion replicate under different enzyme levels, glass bead addition, RPM, and pectin conditions. No values are averaged.

| **Sample ID** | **Day** | **Pectin (mg)** | **Glassbeads (units)** | **Bile/pancrea-tin *** | **RPM **** | **Bioacc. (%)** | **Absolute β-carotene (µg) ***** |
| --- | --- | --- | --- | --- | --- | --- | --- |
| D1F1 PG+100 | 1 | 30 | 10 | Marginal | 100 | 19.99 | 14.99 |
| D1F2 PG+100 | 1 | 30 | 10 | Marginal | 100 | 27.05 | 20.29 |
| D1F3 PG +100 | 1 | 30 | 10 | Marginal | 100 | 23.98 | 17.98 |
| D1F4 PG+100 | 1 | 30 | 10 | Marginal | 100 | 24.89 | 18.67 |
| D1F1 P +100 | 1 | 30 | 0 | Marginal | 100 | 25.82 | 19.37 |
| D1F2 P +100 | 1 | 30 | 0 | Marginal | 100 | 22.14 | 16.60 |
| D1F3 P +100 | 1 | 30 | 0 | Marginal | 100 | 20.29 | 15.22 |
| D1F4 P +100 | 1 | 30 | 0 | Marginal | 100 | 27.65 | 20.74 |
| D1F1 G+ 100 | 1 | 0 | 10 | Marginal | 100 | 26.12 | 19.59 |
| D1F2 G+ 100 | 1 | 0 | 10 | Marginal | 100 | 21.22 | 15.91 |
| D1F3 G+ 100 | 1 | 0 | 10 | Marginal | 100 | 40.23 | 30.17 |
| D1F4 G+ 100 | 1 | 0 | 10 | Marginal | 100 | 42.38 | 31.79 |
| D1F1 + 100 | 1 | 0 | 0 | Marginal | 100 | 28.58 | 21.43 |
| D1F2 + 100 | 1 | 0 | 0 | Marginal | 100 | 29.81 | 22.36 |
| D1F3 + 100 | 1 | 0 | 0 | Marginal | 100 | 24.89 | 18.67 |
| D1F4 + 100 | 1 | 0 | 0 | Marginal | 100 | 21.52 | 16.14 |
| D2F1 PG++100 | 2 | 30 | 10 | Physiological | 100 | 34.56 | 25.92 |
| D2F2 PG++100 | 2 | 30 | 10 | Physiological | 100 | 32.90 | 24.67 |
| D2F3 PG ++100 | 2 | 30 | 10 | Physiological | 100 | 18.78 | 14.08 |
| D2F4 PG++100 | 2 | 30 | 10 | Physiological | 100 | 24.18 | 18.13 |
| D2F1 P ++100 | 2 | 30 | 0 | Physiological | 100 | 19.19 | 14.39 |
| D2F2 P ++100 | 2 | 30 | 0 | Physiological | 100 | 16.28 | 12.21 |
| D2F3 P++100 | 2 | 30 | 0 | Physiological | 100 | 13.79 | 10.34 |
| D2F4 P ++100 | 2 | 30 | 0 | Physiological | 100 | 21.68 | 16.26 |
| D2F1 G++ 100 | 2 | 0 | 10 | Physiological | 100 | 50.36 | 37.77 |
| D2F2 G++ 100 | 2 | 0 | 10 | Physiological | 100 | 47.86 | 35.90 |
| D2F3 G++ 100 | 2 | 0 | 10 | Physiological | 100 | 49.10 | 36.83 |
| D2F4 G++100 | 2 | 0 | 10 | Physiological | 100 | 54.10 | 40.57 |
| D2F1 ++ 100 | 2 | 0 | 0 | Physiological | 100 | 21.68 | 16.26 |
| D2F2 ++ 100 | 2 | 0 | 0 | Physiological | 100 | 26.66 | 20.00 |
| D2F3 ++ 100 | 2 | 0 | 0 | Physiological | 100 | 30.82 | 23.12 |
| D2F4 ++ 100 | 2 | 0 | 0 | Physiological | 100 | 32.07 | 24.05 |
| *D2F1 + 100* | 2 | 0 | 0 | *Marginal (ctrl)* | 100 | 28.54 | 21.40 |
| *D2F2 + 100* | 2 | 0 | 0 | *Marginal (ctrl)* | 100 | 28.33 | 21.25 |
| *D2F3 + 100* | 2 | 0 | 0 | *Marginal (ctrl)* | 100 | 28.75 | 21.56 |
| *D2F4 + 100* | 2 | 0 | 0 | *Marginal (ctrl)* | 100 | 19.19 | 14.39 |
| D3F1 G+++100 | 3 | 0 | 10 | Elevated | 100 | 43.41 | 32.55 |
| D3F2 G+++100 | 3 | 0 | 10 | Elevated | 100 | 47.65 | 35.73 |
| D3F3 G+++100 | 3 | 0 | 10 | Elevated | 100 | 35.52 | 26.64 |
| D3F4 G+++100 | 3 | 0 | 10 | Elevated | 100 | 43.41 | 32.55 |
| D3F1 +++100 | 3 | 0 | 0 | Elevated | 100 | 31.88 | 23.91 |
| D3F2 +++100 | 3 | 0 | 0 | Elevated | 100 | 25.52 | 19.14 |
| D3F3 +++100 | 3 | 0 | 0 | Elevated | 100 | 38.56 | 28.92 |
| D3F4 +++100 | 3 | 0 | 0 | Elevated | 100 | 42.19 | 31.64 |
| D3F1 PG+++100 | 3 | 30 | 10 | Elevated | 100 | 37.04 | 27.78 |
| D3F2 PG+++100 | 3 | 30 | 10 | Elevated | 100 | 24.31 | 18.23 |
| D3F3 PG+++100 | 3 | 30 | 10 | Elevated | 100 | 26.74 | 20.05 |
| D3F4 PG+++100 | 3 | 30 | 10 | Elevated | 100 | 34.62 | 25.96 |
| D3F1 P+++100 | 3 | 30 | 0 | Elevated | 100 | 30.07 | 22.55 |
| D3F2 P+++100 | 3 | 30 | 0 | Elevated | 100 | 37.34 | 28.01 |
| D3F3 P+++100 | 3 | 30 | 0 | Elevated | 100 | 32.19 | 24.14 |
| D3F4 P+++100 | 3 | 30 | 0 | Elevated | 100 | 24.91 | 18.69 |
| *D3F1 + 100* | 3 | 0 | 0 | *Marginal (ctrl)* | 100 | 29.76 | 22.32 |
| *D3F2 + 100* | 3 | 0 | 0 | *Marginal (ctrl)* | 100 | 14.00 | 10.50 |
| *D3F3 + 100* | 3 | 0 | 0 | *Marginal (ctrl)* | 100 | 30.68 | 23.01 |
| *D3F4 + 100* | 3 | 0 | 0 | *Marginal (ctrl)* | 100 | 30.37 | 22.78 |
| D4F1 PG+140 | 4 | 30 | 10 | Marginal | 140 | 12.19 | 9.14 |
| D4F2 PG+140 | 4 | 30 | 10 | Marginal | 140 | 16.67 | 12.50 |
| D4F3 PG +140 | 4 | 30 | 10 | Marginal | 140 | 15.39 | 11.54 |
| D4F4 PG +140 | 4 | 30 | 10 | Marginal | 140 | 15.39 | 11.54 |
| D4F1 P +140 | 4 | 30 | 0 | Marginal | 140 | 13.47 | 10.10 |
| D4F2 P +140 | 4 | 30 | 0 | Marginal | 140 | 21.46 | 16.09 |
| D4F3 P +140 | 4 | 30 | 0 | Marginal | 140 | 13.47 | 10.10 |
| D4F4 P +140 | 4 | 30 | 0 | Marginal | 140 | 16.98 | 12.74 |
| D4F1 G+ 140 | 4 | 0 | 10 | Marginal | 140 | 27.21 | 20.40 |
| D4F2 G+ 140 | 4 | 0 | 10 | Marginal | 140 | 25.29 | 18.97 |
| D4F3 G+ 140 | 4 | 0 | 10 | Marginal | 140 | 26.89 | 20.17 |
| D4F4 G+ 140 | 4 | 0 | 10 | Marginal | 140 | 24.97 | 18.73 |
| D4F1 + 140 | 4 | 0 | 0 | Marginal | 140 | 32.32 | 24.24 |
| D4F2 + 140 | 4 | 0 | 0 | Marginal | 140 | 29.12 | 21.84 |
| D4F3 + 140 | 4 | 0 | 0 | Marginal | 140 | 13.15 | 9.87 |
| D4F4 + 140 | 4 | 0 | 0 | Marginal | 140 | 9.96 | 7.47 |
| D5F1 PG++140 | 5 | 30 | 10 | Physiological | 140 | 29.50 | 22.12 |
| D5F2 PG++140 | 5 | 30 | 10 | Physiological | 140 | 15.68 | 11.76 |
| D5F4 PG++140 | 5 | 30 | 10 | Physiological | 140 | 31.28 | 23.46 |
| D5F3 PG++140 | 5 | 30 | 10 | Physiological | 140 | 41.09 | 30.81 |
| D5F1 P++140 | 5 | 30 | 0 | Physiological | 140 | 30.84 | 23.13 |
| D5F2 P++140 | 5 | 30 | 0 | Physiological | 140 | 36.18 | 27.14 |
| D5F3 P++140 | 5 | 30 | 0 | Physiological | 140 | 25.94 | 19.45 |
| D5F4 P++140 | 5 | 30 | 0 | Physiological | 140 | 39.75 | 29.82 |
| D5F1 G++140 | 5 | 0 | 10 | Physiological | 140 | 44.22 | 33.16 |
| D5F2 G++ 140 | 5 | 0 | 10 | Physiological | 140 | 41.98 | 31.48 |
| D5F3 G++ 140 | 5 | 0 | 10 | Physiological | 140 | 43.32 | 32.49 |
| D5F4 G++140 | 5 | 0 | 10 | Physiological | 140 | 31.28 | 23.46 |
| D5F1++140 | 5 | 0 | 0 | Physiological | 140 | 46.44 | 34.83 |
| D5F2++ 140 | 5 | 0 | 0 | Physiological | 140 | 43.32 | 32.49 |
| D5F3++140 | 5 | 0 | 0 | Physiological | 140 | 41.09 | 30.81 |
| D5F4++140 | 5 | 0 | 0 | Physiological | 140 | 42.43 | 31.82 |
| *D5F1 + 140* | 5 | 0 | 0 | *Marginal (ctrl)* | 140 | 29.50 | 22.12 |
| *D5F2 + 140* | 5 | 0 | 0 | *Marginal (ctrl)* | 140 | 10.32 | 7.74 |
| *D5F3 + 140* | 5 | 0 | 0 | *Marginal (ctrl)* | 140 | 31.28 | 23.46 |
| *D5F4 + 140* | 5 | 0 | 0 | *Marginal (ctrl)* | 140 | 13.46 | 10.09 |
| D6F1 PG+++140 | 6 | 30 | 10 | Elevated | 140 | 22.32 | 16.74 |
| D6F2PG+++140 | 6 | 30 | 10 | Elevated | 140 | 18.15 | 13.61 |
| D6F3PG+++140 | 6 | 30 | 10 | Elevated | 140 | 23.44 | 17.58 |
| D6F4PG+++140 | 6 | 30 | 10 | Elevated | 140 | 22.88 | 17.16 |
| D6F1P+++140 | 6 | 30 | 0 | Elevated | 140 | 16.20 | 12.15 |
| D6F2P+++140 | 6 | 30 | 0 | Elevated | 140 | 17.03 | 12.77 |
| D6F3P+++140 | 6 | 30 | 0 | Elevated | 140 | 20.09 | 15.07 |
| D6F4P+++140 | 6 | 30 | 0 | Elevated | 140 | 18.71 | 14.03 |
| D6F1G+++ 140 | 6 | 0 | 10 | Elevated | 140 | 16.76 | 12.57 |
| D6F2G+++140 | 6 | 0 | 10 | Elevated | 140 | 19.54 | 14.65 |
| D6F3G+++ 140 | 6 | 0 | 10 | Elevated | 140 | 19.82 | 14.87 |
| D6F4G+++140 | 6 | 0 | 10 | Elevated | 140 | 22.60 | 16.95 |
| D6F1 +++ 140 | 6 | 0 | 0 | Elevated | 140 | 16.76 | 12.57 |
| D6F2 +++ 140 | 6 | 0 | 0 | Elevated | 140 | 18.98 | 14.23 |
| D6F3 +++ 140 | 6 | 0 | 0 | Elevated | 140 | 23.16 | 17.37 |
| D6F4 +++ 140 | 6 | 0 | 0 | Elevated | 140 | 24.55 | 18.42 |
| *D6F1 + 140* | 6 | 0 | 0 | *Marginal (ctrl)* | 140 | 24.27 | 18.20 |
| *D6F2 + 140* | 6 | 0 | 0 | *Marginal (ctrl)* | 140 | 18.71 | 14.03 |
| *D6F3 + 140* | 6 | 0 | 0 | *Marginal (ctrl)* | 140 | 21.48 | 16.11 |
| *D6F4 + 140* | 6 | 0 | 0 | *Marginal (ctrl)* | 140 | 20.09 | 15.07 |

* Bile/pancreatin ratio: “Marginal”: 3.4 mg/mL bile, 100 U/mL pancreatin; “Physiological”: 6.8 mg/mL bile, 200 U/mL pancreatin; “Elevated”: 10.3 mg/mL bile, 300 U/mL pancreatin.

** Rotations per minute of shaking water bath.

*** Absolute β-carotene (µg) = bioaccessibility (%) × 75 µg β-carotene added per digestion / 100. Bioaccessibility normalised to the daily Marginal/no-pectin/no-glassbead control set to 100%; control replicates are shaded.

**Supplementary Table 3-** Mean zeta potential (mV) ± SD from 3 replicates under different enzyme levels, glass bead addition, RPM, and pectin conditions. Given are estimated marginal means and pooled standard deviations as obtained from linear mixed models.

| **Pectin (mg)** | **Glassbeads (numbers)** | **Bile/pancreatin*** | **RPM**** | **Mean** | **SD** |
| --- | --- | --- | --- | --- | --- |
| 0 | 0 | Elevated | 100 | 28.1 | 3.5 |
| 0 | 0 | Elevated | 140 | 29.7 | 6.3 |
| 0 | 0 | Marginal | 100 | 53.8 | 3.4 |
| 0 | 0 | Marginal | 140 | 51.1 | 0.7 |
| 0 | 0 | Physiological | 100 | 47.0 | 5.2 |
| 0 | 0 | Physiological | 140 | 51.2 | 2.2 |
| 0 | 10 | Elevated | 100 | 30.8 | 9.0 |
| 0 | 10 | Elevated | 140 | 28.9 | 4.5 |
| 0 | 10 | Marginal | 100 | 52.2 | 2.8 |
| 0 | 10 | Marginal | 140 | 51.3 | 2.0 |
| 0 | 10 | Physiological | 100 | 49.6 | 1.9 |
| 0 | 10 | Physiological | 140 | 38.2 | 13.8 |
| 30 | 0 | Elevated | 100 | 25.8 | 6.1 |
| 30 | 0 | Elevated | 140 | 28.9 | 2.0 |
| 30 | 0 | Marginal | 100 | 53.3 | 1.6 |
| 30 | 0 | Marginal | 140 | 50.8 | 2.7 |
| 30 | 0 | Physiological | 100 | 43.8 | 3.8 |
| 30 | 0 | Physiological | 140 | 41.7 | 4.9 |
| 30 | 10 | Elevated | 100 | 19.1 | 3.9 |
| 30 | 10 | Elevated | 140 | 20.5 | 6.2 |
| 30 | 10 | Marginal | 100 | 52.0 | 3.2 |
| 30 | 10 | Marginal | 140 | 50.4 | 2.8 |
| 30 | 10 | Physiological | 100 | 42.4 | 0.2 |
| 30 | 10 | Physiological | 140 | 42.1 | 6.8 |

* Bile/pancreatin ratio —

- “Marginal” refers to the lowest tested bile (3.4 mg/mL) and pancreatin (100 U/mL) concentrations.
- “Physiological” corresponds to medium bile (6.8 mg/mL) and pancreatin (200 U/mL) concentrations.
- “Elevated” indicates the highest tested bile (10.3 mg/mL) and pancreatin (300 U/mL) concentrations.

** Revolutions per minute of shaking water bath.

**Supplementary Table 4 .** A table of fixed effects from the full linear mixed model for β-carotene bioaccessibility, including all main effects and interaction terms. Non-significant interaction terms (subsequently removed from the final reduced model) are retained here for transparency.

| **Source** | **Numerator df** | **Denominator df** | **p-value** |
| --- | --- | --- | --- |
| Intercept | 1 | 88 | **<0.001** |
| Pectin | 1 | 88 | **<0.001** |
| Glass beads | 1 | 88 | **0.001** |
| Bile/pancreatin | 2 | 88 | **<0.001** |
| RPM | 1 | 88 | **<0.001** |
| Pectin × Glass beads | 1 | 88 | **0.032** |
| Pectin × Bile/pancreatin | 2 | 88 | **0.004** |
| Pectin × RPM | 1 | 88 | 0.125 |
| Glass beads × Bile/pancreatin | 2 | 88 | 0.281 |
| Glass beads × RPM | 1 | 88 | **<0.001** |
| Bile/pancreatin × RPM | 2 | 88 | **<0.001** |
| Pectin × Glass beads × Bile/pancreatin | 2 | 88 | 0.624 |
| Pectin × Glass beads × RPM | 1 | 88 | 0.067 |
| Pectin × Bile/pancreatin × RPM | 2 | 88 | 0.145 |
| Glass beads × Bile/pancreatin × RPM | 2 | 88 | **0.002** |
| Pectin × Glass beads × Bile/pancreatin × RPM | 2 | 88 | 0.393 |

*Dependent variable: β-carotene bioaccessibility . Bold p-values indicate statistical significance*

*(p < 0.05). Bile/pancreatin = enzyme/bile concentration level (marginal, physiological, elevated).*
